# Power and sample size calculation for non-inferiority trials with treatment switching in intention-to-treat analysis comparing RMSTs

**DOI:** 10.21203/rs.3.rs-5418253/v1

**Published:** 2024-12-12

**Authors:** Austin Shih, Chih-Yuan Hsu, Yu Shyr

**Affiliations:** 1 Department of Mathematics, Vanderbilt University, Nashville, TN, USA; 2 Department of Biostatistics, Vanderbilt University Medical Center, Nashville, TN, USA; 3 Center for Quantitative Sciences, Vanderbilt University Medical Center, Nashville, TN, USA

**Keywords:** crossover, intention-to-treat analysis, non-inferiority trials, restricted mean survival time

## Abstract

**Background:**

Difference in Restricted Mean Survival Time (DRMST) has attracted attention and is increasingly used in non-inferiority (NI) trials because of its superior power in detecting treatment effects compared to hazard ratio. However, when treatment switching (also known as crossover) occurs, the widely used intention-to-treat (ITT) analysis can underpower or overpower NI trials.

**Methods:**

We propose a simulation-based approach, named *nifts*, to calculate powers and determine the necessary sample size to achieve a desired power for non-inferiority trials that allow treatment switching, in ITT analysis using DRMST.

**Results:**

Real-world and simulated examples are used to illustrate the proposed method and examine how switching probability, switching time, the relative effectiveness of treatments, allocation ratio, and even time distribution influence powers and sample sizes. Our simulation study shows that switching time and switching probability decrease or increase powers and sample sizes compared to those in the scenarios without treatment switching. A shorter switching time and a higher switching probability amplify the magnitude of these changes. The direction of the change in powers and sample sizes depends on the relative effectiveness of the treatments. When $m_{2}/m_{1}>1$, power decreases and sample size increases, while $m_{2}/m_{1}<1$ leads to the opposite effect, where $m_{1}$ and $m_{2}$ are the median survivals in the control and experimental groups, respectively.

**Conclusions:**

This simulation-based approach offers a preview of how treatment switching can influence powers and sample sizes in NI trials, providing investigators with useful information before conducting the trials. *nifts* is freely available at https://github.com/cyhsuTN/nifts.

## 1 Introduction

A randomized controlled trial (RCT) is regarded as the gold standard for assessing the effectiveness of new treatments. Among the various types of RCTs, a non-inferiority (NI) trial aims to demonstrate that a new treatment is not significantly worse than an existing one, while potentially offering additional benefits such as fewer side effects or lower costs. One increasingly popular approach for evaluating treatment effects in NI trials with time-to-event outcomes is to compare restricted mean survival times (RMST) between treatment groups [1–3]. RMST provides a straightforward summary by averaging survival times up to a specified time point [4] and does not rely on the proportional hazards (PH) assumption, which is frequently violated in clinical trials [5]. As a result, Royston and Parmar [6] suggested using the difference in RMSTs (DRMST) between treatment groups as an alternative to the hazard ratio (HR) for designing randomized trials with time-to-event outcomes, including power and sample size calculations. Furthermore, DRMST has greater power in detecting treatment effects compared to HR, even under the PH assumption [7, 8]. Methods for determining powers and sample sizes in NI trials using DRMST have been proposed [9, 10].

In RCTs, including NI trials, treatment switching from the control group to the experimental group may occur due to ethical concerns or other reasons [11, 12]. This switch may happen when a disease progresses, when healthcare providers believe the patient’s prognosis will improve with the experimental treatment, or when patients prefer the new treatment due to perceived benefits such as fewer side effects or greater convenience [11, 13]. However, treatment switching can confound the results of intention-to-treat (ITT) analysis, making it difficult to determine the true treatment effect. ITT analysis includes all participants with randomization and compares their responses to determine the treatment effect according to the initially assigned treatment groups, regardless of what treatment they received. This may potentially lead to underpowered trials and inconclusive results [12]. An alternative approach is per-protocol analysis that excludes participants who switch treatments from the analysis. Nevertheless, this can heavily bias the results if there is a significant difference in prognosis between the included and excluded participants, particularly if the treatment switching is associated with prognostic variables [14]. Therefore, ITT analysis is still often used in the final analysis. Deng et al. [15] proposed a simulation-based approach to preview power reduction and sample sizes required in superiority trials with treatment switching in ITT analysis using the logrank test.

In this study, we propose a simulation-based approach, named *nifts*, to determine power and sample size in NI trials that involve treatment switching when comparing RMSTs between two treatment groups in ITT analysis. To accelerate the computation of sample sizes, a monotonic smoothing technique is employed to estimate the power trend as sample sizes increase [16]. We utilize both real-world and simulated examples to illustrate the proposed method and examine how switching probability, switching time, the relative effectiveness of treatments, allocation ratio, and even time distribution influence power and sample sizes. *nifts* is freely available at https://github.com/cyhsuTN/nifts.

## 2 Methods

### 2.1 Non-inferiority Trials using DRMST

Denote the survival functions for the control group and the experimental group by $S_{1}(t)$ and $S_{2}(t)$, respectively. The restricted mean survival times (RMST) at a specified time τ(τ>0) for the two groups are defined as $R_{i}(\tau )=\int_{0}^{\tau }S_{i}(t)dt,i=1\;\text{and}\;2$. The difference in RMSTs between the two groups (DRMST) is given by $\Delta (\tau )=R_{2}(\tau )-R_{1}(\tau )$. The estimate for Δ(τ) is $\hat{\Delta }(\tau )={\overset{ˆ}{R}}_{2}(\tau )-{\hat{R}}_{1}(\tau )$, where ${\hat{R}}_{i}(\tau )=\int_{0}^{\tau }{\overset{ˆ}{S}}_{i}(t)dt$ and ${\overset{ˆ}{S}}_{i}(t)$ is the Kaplan-Meier estimate for $S_{i}(t)$. The aim of a non-inferiority trial using DRMST is to test $H_{0}:\Delta (\tau )\leq -\delta$ vs $H_{1}:\Delta (\tau )>-\delta$, where δ>0 is a prespecified margin. If

$$\hat{\Delta }(\tau )-z_{1-\alpha }\text{SE}(\hat{\Delta }(\tau ))>-\delta ,$$

we reject the null hypothesis at a one-sided significance level of α and claim that non-inferiority holds (i.e., the experimental treatment is not significantly worse than the control). Here, $z_{1-\alpha }$ represents the (1-α) th quantile of the standard normal distribution, and $\text{SE}(\hat{\Delta }(\tau ))=\left.\sqrt{\left.\left.\text{Var}\hat{\left({\hat{R}}_{1}\right.}(\tau )\right)+\text{Var}\hat{\left({\hat{R}}_{2}\right.}(\tau )\right)}\cdot \text{Var}\hat{\left({\hat{R}}_{l}\right.}(\tau )\right)$ is the estimate for the variance of ${\overset{ˆ}{R}}_{i}(\tau )$, whose explicit expression can be found in [9]. Both ${\overset{ˆ}{R}}_{i}(\tau )$ and $\left.\text{Var}\hat{\left({\overset{ˆ}{R}}_{l}\right.}(\tau )\right)$ can be calculated using the survfit function in the *survival* R package.

### 2.2 The Choice of Margins

In this study, we propose three options for selecting margins.

#### Preserved fraction of the RMST of the control group

We aim for $R_{2}(\tau )$ to maintain at least the preserved fraction, $f_{1}$, of the RMST of the control group, where $0<f_{1}<1$. This means $R_{2}(\tau )>f_{1}R_{1}(\tau )$. Thus, $\Delta (\tau )>-\left(1-f_{1}\right)R_{1}(\tau )$ and $\delta =(1-\left.f_{1}\right)R_{1}(\tau )$.

#### Preserved fraction of the DRMST between the control and the placebo groups

In this option, we aim for the RMST of the experimental group to be better than the RMST of the placebo group, and the DRMST between the experimental and placebo groups to maintain at least the preserved fraction, $f_{2}$, of the DRMST between the control and placebo groups, where $0<f_{2}<1$. This means $R_{1}(\tau )-R_{0}(\tau )>0$ and $R_{2}(\tau )-R_{0}(\tau )>f_{2}\left(R_{1}(\tau )-R_{0}(\tau )\right)$, where $R_{0}(\tau )=\int_{0}^{\tau }S_{0}(t)dt$ and $S_{0}(t)$ is the survival function for the placebo group. Typically, $R_{1}(\tau )-R_{0}(\tau )>0$ holds, so $\delta =\left(1-f_{2}\right)\left(R_{1}(\tau )-R_{0}(\tau )\right)$.

#### Conversion from the hazard ratio

Given $S_{1}(t)$ and assuming proportional hazards, a margin (1/θ) for the hazard ratio (HR) of the experimental group to the control group can be converted to a margin for DRMST from ${\text{HR}}_{21}<1/\theta$ to Δ(τ)>-δ, where $\delta =R_{1}(\tau )-R_{\theta }(\tau )$ with $R_{\theta }(\tau )=\int_{0}^{\tau }{\left(S_{1}(t)\right)}^{1/\theta }dt$ and 0<θ<1.

### 2.3 The Design Setting and Assumption

**Figure 1:**
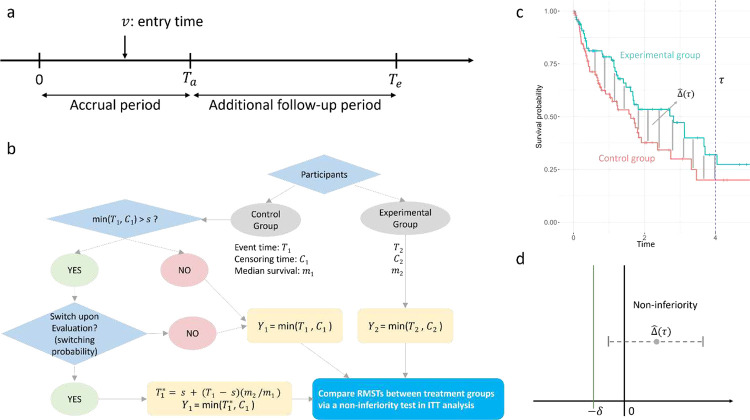
An overview of *nifts*. (a) Accrual time and trial duration. (b) Treatment switching. (c) Difference in RMSTs between two treatment groups. (d) Non-inferiority holds if the lower bound of DRMST is larger than -δ.

Denote the trial duration by $T_{e}>0$ and the accrual time during which participants are recruited by $T_{a}\geq 0$. $T_{e}-T_{a}\geq 0$ is the additional follow-up time. Participants are assumed to enter the study uniformly during the accrual period, i.e., $v\sim U\left(0,T_{a}\right)$, where v is the entry time of a participant (Figure 1a). If $T_{a}=0$, all participants are assumed to enter the study at its start. We assume participants are randomly assigned to the control group or the experimental group with an allocation ratio of r, where r is defined as the ratio of the participants in the experimental group to those in the control group.

Denote the survival times for participants in the control and experimental groups by $T_{1}$ and $T_{2}$, respectively, and assume $T_{1}$ and $T_{2}$ follow Weibull distributions with the same shape parameter but different scale parameters. The scale and shape parameters of the two Weibull distributions are determined by given median survivals of $m=m_{1}$ and $m_{2}$, and a survival rate at a specific time t in the control group. Specifically, the scale and shape parameters are obtained by solving the equations: scale $(\text{log}2{)}^{1/\text{shape}}=m$ and $\text{exp}\left(-(t/\text{scale}{)}^{\text{shape}}\right)=$ survival rate.

Denote the censoring times for participants in the control and experimental groups by $C_{1}$ and $C_{2}$, defined as the duration from randomization to either dropping out of the trial or reaching the end of the trial if participants don’t experience the event of interest. Therefore, the censoring time comprises dropout censoring and administrative censoring, and its distributions can be formulated as follows [17]:

$$f\left(c|v\right)=d\left(c\right)I\left(0<c<T_{e}-v\right)+\overset{‾}{D}\left(T_{e}-v\right)I\left(c=T_{e}-v\right),$$

where d(c) and $\overset{‾}{D}(c)$ are the density function and survival function of the dropout censoring, respectively. I(⋅) is the indicator function. The dropout censoring is assumed to follow a uniform distribution U(0,h), where h is determined by a given censoring rate of the control group under no treatment switching (see Supplementary Materials for details). For the scenario of no dropout censoring, we set $P\left(c=T_{e}-v|v\right)=1$. Additionally, the distributions of the censoring times in the two groups are assumed to be the same.

### 2.4 Treatment Switching

*nifts* allows participants in the control group to switch to the experimental group if certain predetermined conditions are met. For example, if patients with cancer have a disease progression before death (assume death is the event of interest), they may switch from the standard treatment to the new treatment after disease progression and evaluation by the investigators [11]. Denote the switching time by s, defined as the duration from randomization to the moment when a participant may switch, with a probability $p_{s}$. The switching probability $\left(p_{s}\right)$ is the likelihood that a participant who qualifies for treatment switching will switch from the control group to the experimental group after evaluation by healthcare professionals.

Five options for the distributions of the switching time are provided (Table 1), as used in [15]. The first three options assume s is correlated with $T_{1}$, while the other two options assume s is not correlated with $T_{1}$. The parameters in the assumed distributions are determined based on the given values of $r_{s}$ and $\rho_{s}$ (See Supplementary Materials for details). $r_{s}=E(s)/E\left(T_{1}\right)$ denotes the ratio of the average switching time to the average survival time of the control group, and $\rho_{s}$ denotes the correlation between s and $T_{1}$.

The survival time for participants starting from switching is assumed to increase by $m_{2}/m_{1}$, based on the rank preserving structural failure time model (RPSFTM) [18]. Thus, the survival time of the participants with treatment switching will be $T_{1}^{*}=s+\left(T_{1}-s\right)\times \left(m_{2}/m_{1}\right)$. Therefore, the observable survival time $Y_{1}$ for the participants without and with treatment switching from the control group to the experimental group will be $\text{min}\left(T_{1},C_{1}\right)$ and $\text{min}\left(T_{1}^{*},C_{1}\right)$, respectively. The observable survival time $Y_{2}$ for the participants in the experimental group will be $\text{min}\left(T_{2},C_{2}\right)$. Finally, a non-inferiority test for DRMST between the two samples $\left\{Y_{1}\right\}$ and $\left\{Y_{2}\right\}$ in ITT analysis is performed (Figure 1b–1d).

### 2.5 The Proposed Method

The proposed *nifts* includes two main functions: *calculate_power* and *calculate_size*. The first function calculates power and outputs the associated expected number of events in the control and experimental groups. The latter determines the required sample size to achieve a specified power. The required sample size is obtained by a monotonically increasing power curve to the sample sizes. This curve is estimated using a monotonic smoothing technique [16] based on a finite number of power points and sample sizes.

The *calculate_power* function includes 21 parameters to simulate various scenarios: $n,r,m_{1},m_{2}$, *shape*, $f_{1},m_{0},f_{2}$, *margin*, $p_{s},r_{s},\rho_{s}$, *s.dist*, *censoring.rate*, $T_{a},T_{e},\tau$, *one.sided.alpha*, *TXswitch*, *n_simulations*, and *seed*. n : sample size of the control group, r : allocation ratio, $m_{1}$ and $m_{2}$ : median survivals, *shape*: shape parameter of the Weibull distributions for event times, $f_{1}$ and $f_{2}$ : preserved fractions, $m_{0}$ : median survival of the placebo group for calculating $R_{0}(\tau )$ if $f_{2}$ is given, margin: non-inferiority *margin*, $p_{s}$ : switching probability, $r_{s}$ : ratio of E(s) to $E\left(T_{1}\right),\rho_{s}$ : correlation of s and $T_{1}$, *s.dist*: options for the distributions of switching time (*s.dist* = “unif”, “beta”, “gamma”, “indepExp”, or a numeric value), *censoring.rate*: censoring rate of the control group (*censoring.rate* = “AC.only” meaning administrative censoring only, or = a numeric value), $T_{a}$ and $T_{e}$: accrual duration and trial duration, τ : prespecified time for RMST calculation, *one.sided.alpha*: one-sided significance level, *TXswitch*: direction of treatment switching (*TXswitch* = “1to2” (default) or “2to1”), *n_simulations*: number of simulations, and *seed*: simulation seed. When $f_{1}$ is provided, the first margin option is used. When $f_{2}$ and $m_{0}$ are provided, the second margin option is used. A customized margin is applied when a numeric *margin* is provided, for example, an RMST margin converted from an HR margin.

The *calculate_size* function uses the same parameters as *calculate_power* while adding 4 parameters $n_{L},n_{U},B$, epwr. The lower $\left(n_{L}\right)$ and upper $\left(n_{U}\right)$ bounds are minimum and maximum sample sizes users input when exploring sample sizes for a desired expected power (*epwr*). The function divides the range of the bounds into B equal intervals and calculate the powers at $n=n_{L}+k\times w$, where $w=\text{round}\left(\left(n_{U}-n_{L}\right)/B\right)$ and k=0,1,2,…,B. A shape constrained additive model [16] is employed to fit a monotonically increasing power curve to the sample sizes, from which the required sample size is determined.

## 3 Results

### 3.1 Parameters Setting via Real-World Examples

The first example is an open-label phase III trial comparing survival benefits in patients with chemotherapy-refractory metastatic colorectal cancer, who were randomly assigned to either panitumumab + best supportive care (BSC) or BSC alone [11]. A total of 231 patients were randomly assigned to panitumumab + BSC, and 232 to BSC alone. Among the BSC alone patients, 85% experienced disease progression, and 76% switched to panitumumab + BSC after evaluation by the investigator. Thus, the switch probability $p_{s}$ was 0.89 (= 0.76/0.85). We use the trial scenario (ClinicalTrial.gov: NCT00113763) to illustrate the proposed method for power and sample size calculation in NI trials with treatment switching when using DRMST in ITT analysis. We set $T_{a}=0,T_{e}=26$ (in months), n=232 with a 1:1 allocation ratio (r=1), and a censoring rate of 0.05 for BSC alone group. We compare the RMSTs of overall survival at τ=12 (in months) between the two groups with a preserved fraction of $f_{1}=0.8$ (i.e., $\text{margin}=0.2R_{1}(\tau )$), and assume Weibull distributions with $m_{1}=6.0$ and $m_{2}=6.4$ and shape =1 for event time. If there were no treatment switching, the power at n=232 could reach 90% at a one-sided significance level of 0.005 in this setting.

Next, we examine the changes in powers and required sample sizes when treatment switching occurs with a switch probability of $p_{s}=0.89$. We assume *s.dist* = “gamma” or “indepExp” with $r_{s}=0.3\left(=1.96/6.4\right.$, the ratio of E(s)=1.96 (the reported mean PFS) to $\left.E\left(T_{1}\right)=6.4\right)$. For *s.dist* = “gamma”, we assume $\rho_{s}=0.1,0.3,0.5,0.7$, and 0.9 to model low to high correlations between progression-free survival and overall survival. The resulting powers at n=232 range from 0.775 to 0.833, which are less than 0.9, and the required sample sizes to achieve the power of 0.9 range from 284 to 308 (Table 2).

The second example is a non-inferiority trial involving 1,234 women with early-stage breast cancer who have undergone breast-conserving surgery [13]. This trial compares hypofractionated radiotherapy to standard radiotherapy for preventing local recurrence of invasive breast cancer. Between April 1993 and September 1996, 622 and 612 patients were randomly assigned to hypofractionated radiotherapy and standard radiotherapy, respectively, and were followed up to 12 years $\left(T_{a}=3.5,T_{e}=12\right.$, and r=1) with 7.9% dropout censoring. Among the patients randomized to hypofractionated radiotherapy, 1.2% selected standard radiotherapy instead ($p_{s}=0.012,s=0$, and *TXswitch* = “2tol”) [9]. Given the assumption of a 7% 5-year local recurrence rate for standard radiotherapy [13], we assume Weibull distributions with $m_{1}=m_{2}=-5\text{log}(2)/\text{log}(0.93)=47.8$ and *shape* = 1 for event time, and a dropout censoring rate of 4% (about a half of 7.9%) for standard radiotherapy, i.e., *censoring.rate* = 0.902 (including 86.2% administrative censoring). Based on the hypofractionated radiotherapy is not worse than the standard radiotherapy by 5% in local recurrence-free survival at 5 years, the HR margin is 1/θ=log⁡(0.88)/log⁡(0.93)=1.762.

We compare the RMSTs at τ=5.75 and 10 (corresponding to two analysis times in [13]) between the two radiotherapy groups. The DRMST margins, converted from the HR margin, are 0.169 and 0.484, respectively. With a one-sided significance level of 0.05 and a power of 0.9, the required sample sizes n in the standard radiotherapy group are 550 for τ=5.75 and 376 for τ=10.

### 3.2 Simulation Scenarios

Various simulations are conducted to examine the impact of treatment switching on power and sample size estimation in NI trials using DRMST in ITT analysis. These simulations consider different relative effectiveness of the experimental versus the control group ($m_{2}/m_{1}=1.1$ and 0.9), switching times ($r_{s}=0.5$ and 0.25) and switching probabilities ($p_{s}=0.2$ and 0.4), event time distributions (Weibull distributions with *shape* = 1,0.75, and 1.25), distributions of switching time (*s.dist* = “unif”, “beta”, “gamma”, and “indepExp”), and allocation ratios (r=1 and 2). For each scenario, we set $T_{a}=3,T_{e}=5,\tau =5,m_{1}=1,\rho_{s}=0.775$, *censoring.rate* = 0.2, and *n_simulations* = 5000. Also, $m_{0}=0.5$ and $f_{2}=0.5$ are used for calculating $R_{0}(\tau )$ and the DRMST margin, i.e., the margin equals $0.5\left(R_{1}(\tau )-R_{0}(\tau )\right)$. When $r_{s}=0.5,\rho_{s}=0.775$, and event times follow exponential distributions, the results of assuming *s.dist* = “beta” will be similar to those of assuming *s.dist* = “unif” because the shape1 and shape 2 parameters in the beta distributions are close to 1. The one-sided significance level is set at 0.025.

#### Effect of relative effectiveness on power and sample size

Treatment switching results in a decrease in power when $m_{2}>m_{1}\left(m_{2}/m_{1}=1.1\right)$ and an increase when $m_{2}<m_{1}\left(m_{2}/m_{1}=0.9\right)$. Consequently, this corresponds an increase and decrease in the ratio $\left(n/n_{ns}\right)$ of sample sizes with treatment switching (n) to those without switching $\left(n_{ns}\right)$, respectively (Table 3). For example, at $p_{s}=0.2$ and *s.dist* = “unif”, the power is 0.776 at $n_{ns}=158$ and $n/n_{ns}=1.044$ when $m_{2}/m_{1}=1.1$, while the power is 0.859 at $n_{ns}=656$ and $n/n_{ns}=0.886$ when $m_{2}/m_{1}=0.9$. Similar changes in powers and sample sizes are observed for other distributions of switching time. The powers decrease to between 0.764 and 0.783 when $m_{2}/m_{1}=1.1$ and increase to between 0.849 and 0.875 when $m_{2}/m_{1}=0.9$. The ratios of sample sizes increase to between 1.019 and 1.082 when $m_{2}/m_{1}=1.1$ and decrease to between 0.849 and 0.886 when $m_{2}/m_{1}=0.9$.

#### Effect of switching probability on power and sample size

When $p_{s}$ increases to 0.4, the magnitude of changes in powers and sample sizes increases. Across four distributions of switching time, when $m_{2}/m_{1}=1.1$, the powers decrease to a range of 0.739 and 0.757, and when $m_{2}/m_{1}=0.9$, the power increase to a range of 0.879 and 0.907. The ratios of sample sizes increase to a range of 1.127 and 1.184 when $m_{2}/m_{1}=1.1$ and decrease to a range of 0.706 and 0.788 when $m_{2}/m_{1}=0.9$.

#### Effect of switching time on power and sample size

When $r_{s}$ is reduced from 0.5 to 0.25, indicating a shorter switching time, the magnitude of changes in powers and sample sizes increases (Table 4). Comparing the results at $p_{s}=0.4$ in Table 4 with those above, across the three distributions of switching time, when $m_{2}/m_{1}=1.1$, the powers decrease to a range of 0.720 and 0.726, and when $m_{2}/m_{1}=0.9$, the power increase to a range of 0.915 and 0.929. The ratios of sample sizes rise to a range of 1.203 and 1.228 when $m_{2}/m_{1}=1.1$ and fall to a range of 0.671 and 0.698 when $m_{2}/m_{1}=0.9$.

We also adjust the shape parameters in Weibull distributions to assess the impact of different event time distributions (Supplementary Figure s1). The changes in powers are similar and there is no significant trend (Supplementary Tables s1, s2, and Table 3). The ratios of sample sizes slightly increase and decrease with the shape values when $m_{2}/m_{1}=1.1$ and 0.9, respectively, except for *s.dist* = “indepExp”. However, the required sample sizes vary significantly, decreasing with the shape values. In addition, when we change the allocation ratio from 1 to 2, the change patterns are similar, but more total sample sizes (n(r+1)) are needed (Supplementary Table s3).

## 4 Discussion

Our simulation study shows that switching time and switching probability can decrease or increase power and sample sizes compared to those in the scenarios without treatment switching. A shorter switching time and a higher switching probability amplify the magnitude of these changes. Whether power and sample sizes decrease or increase depends on the relative effectiveness. When $m_{2}/m_{1}>1$, powers decrease and sample sizes increase, while $m_{2}/m_{1}<1$ leads to the opposite result. When $m_{1}=m_{2}$, treatment switching does not impact power and sample sizes. The changes in powers and sample sizes are not sensitive to the choice of the distributions of switch time. To accelerate the computation of sample sizes, we employ a monotonic smoothing technique [16] to model the power trend as sample sizes increase. The powers at the sample size estimated by the power curve exhibit a bias of less than 2% from the expected power.

*nifts* assumes the effects of the experimental treatment are the same (common treatment effect, made by RPSFTM [18]) for participants initially in the experimental group and those who switch from the control group to the experimental group. This assumption may be problematic, as participants who switch from the control group to the experimental group may have worse survival outcomes. Properly adjusting the accelerated factor $m_{2}/m_{1}$ could help fit the scenario. Multiplying $m_{2}/m_{1}$ by a constant less than 1 might be a solution, but determining this constant value before clinical trials is challenging, even with information from previous similar studies.

In this study, we assume event times follow Weibull distributions rather more flexible distributions like generalized gamma distributions that can fit more real-world scenarios. This is because determining the three parameters for the latter can be challenging for investigators. Besides, median survival times and hazard ratios are still commonly used for power and sample size calculations, so we ultimately choose Weibull distributions that satisfy the proportional hazards assumption. *nifts* will help users calculate the scale and shape parameters required for Weibull distributions when provided the median survivals of two treatment groups and a survival rate at a specific time in the control group.

## 5 Conclusions

We propose a simulation-based approach, *nifts*, for power and sample size calculation in NI trials with treatment switching when comparing the RMSTs of two treatment groups in ITT analysis. This approach offers a preview of how treatment switching can influence powers and sample sizes in NI trials, providing investigators with useful information before conducting the trials.

## Figures and Tables

**Table 1.** Five options for the distributions of switching time are provided.

	Options	Property
s is correlated with $T_{1}$ Assume $s=XT_{1}$ and X is independent of $T_{1}$	X~U(0,1)	$s<T_{1}$
	$X\sim \text{Beta}({\text{shape}}_{1}=a,{\text{shape}}_{2}=b)$ $r_{s}=a/\left(a+b\right)$ and $\rho_{s}=\left\{\frac{a}{a+b}\sqrt{\text{Var}\left(T_{1}\right)}\right\}/{\left\{{\left(\frac{a}{a+b}\right)}^{2}\text{Var}\left(T_{1}\right)+\frac{ab}{{\left(a+b\right)}^{2}\left(a+b+1\right)}E\left(T_{1}^{2}\right)\right\}}^{1/2}$	$s<T_{1}$
	X~Gamma(shape=a,rate=b) $r_{s}=a/b\;\text{and}\;\rho_{s}=\left\{\frac{a}{b}\sqrt{\text{Var}\left(T_{1}\right)}\right\}/{\left\{{\left(\frac{a}{b}\right)}^{2}\text{Var}\left(T_{1}\right)+\frac{a}{b^{2}}E\left(T_{1}^{2}\right)\right\}}^{1/2}$	
s is not correlated with $T_{1}$	s~Exponential(rate=b) $b={\left(r_{s}E\left(T_{1}\right)\right)}^{-1}$	
	s is a specific time. e.g., s=0 denotes the switch occurs at the start of the study	

**Table 2.** Powers and required sample sizes in a NI trial allowing treatment switching with a switch probability of $p_{s}=0.89$ when using DRMST in ITT analysis.

	*s.dist*= “gamma”					*s.dist* = “indepExp”
$\rho_{s}$	0.1	0.3	0.5	0.7	0.9	0
Power at a one-sided significance level of 0.005 with n=232 and r=1	0.775	0.807	0.808	0.815	0.808	0.833
Required sample sizes (n) to achieve the power of 0.9 at the one-sided significance level of 0.005	308	299	293	290	292	284

**Table 3.** Required sample sizes (n) and powers at $n_{ns}$ with $r_{s}=0.5$, shape = 1, and r=1, where $n_{ns}$ denotes the sample size under no treatment switching, given a power of 0.8 and a one-sided significance level of 0.025. E1 and E2 are the expected number of events in the control and experimental groups.

$m_{2}/m_{1}=1.1$ $n_{ns}=158;\text{E}1=126.4;\text{E}2=122.5$		*s.dist*			
		unif	beta	gamma	indepExp
$p_{s}=0.2$	n	165	171	161	170
	E1	131.6	136.4	128.4	135.4
	E2	127.9	132.6	124.8	131.8
	$n/n_{ns}$	1.044	1.082	1.019	1.076
	Power at $n_{ns}$	0.776	0.783	0.775	0.764
	Power at n	0.790	0.813	0.782	0.795
$p_{s}=0.4$	n	178	179	179	187
	E1	141.5	142.3	142.2	148.3
	E2	138.1	138.9	138.8	145.1
	$n/n_{ns}$	1.127	1.133	1.133	1.184
	Power at $n_{ns}$	0.757	0.753	0.745	0.739
	Power at n	0.789	0.801	0.802	0.801
$m_{2}/m_{1}=0.9$ $n_{ns}=656;\text{E}1=524.8;\text{E}2=541.0$		*s.dist*			
		unif	beta	gamma	indepExp
$p_{s}=0.2$	n	581	581	578	557
	E1	466.2	466.3	464.0	447.6
	E2	479.4	479.3	476.8	459.5
	$n/n_{ns}$	0.886	0.886	0.881	0.849
	Power at $n_{ns}$	0.859	0.851	0.849	0.875
	Power at n	0.807	0.801	0.810	0.799
$p_{s}=0.4$	n	513	517	508	463
	E1	412.9	416.2	409.0	373.7
	E2	423.2	426.5	419.1	381.9
	$n/n_{ns}$	0.782	0.788	0.774	0.706
	Power at $n_{ns}$	0.879	0.885	0.899	0.907
	Power at n	0.806	0.805	0.796	0.782

**Table 4.** Required sample sizes (n) and powers at $n_{ns}$ with $r_{s}=0.25$, *shape* = 1 and r=1, where $n_{ns}$ denotes the sample size under no treatment switching, given a power of 0.8 and a one-sided significance level of 0.025. E1 and E2 are the expected number of events in the control and experimental groups.

$m_{2}/m_{1}=1.1$ $n_{ns}=158;\text{E}1=126.4;\text{E}2=122.5$		*s.dist*			
		unif	beta	gamma	indepExp
$p_{s}=0.2$	n	-	178	167	172
	E1	-	141.7	133.1	136.9
	E2	-	138.2	129.4	133.4
	$n/n_{ns}$	-	1.127	1.057	1.089
	Power at $n_{ns}$	-	0.763	0.764	0.759
	Power at n	-	0.804	0.788	0.802
$p_{s}=0.4$	n	-	193	190	194
	E1	-	152.9	150.6	153.6
	E2	-	149.8	147.3	150.5
	$n/n_{ns}$	-	1.222	1.203	1.228
	Power at $n_{ns}$	-	0.720	0.722	0.722
	Power at n	-	0.800	0.806	0.797
$m_{2}/m_{1}=0.9$ $n_{ns}=656;\text{E}1=524.8;\text{E}2=541.0$		*s.dist*			
		unif	beta	gamma	indepExp
$p_{s}=0.2$	n	-	546	539	536
	E1	-	438.8	433.3	431.1
	E2	-	450.4	444.6	442.2
	$n/n_{ns}$	-	0.832	0.822	0.817
	Power at $n_{ns}$	-	0.860	0.865	0.878
	Power at n	-	0.789	0.807	0.809
$p_{s}=0.4$	n	-	458	453	440
	E1	-	370.0	365.9	355.7
	E2	-	377.8	373.7	362.9
	$n/n_{ns}$	-	0.698	0.691	0.671
	Power at $n_{ns}$	-	0.915	0.929	0.924
	Power at n	-	0.815	0.800	0.788

- *s.dist* = “unif” does not satisfy $r_{s}=0.25$.

## Data Availability

Additional file 1: Supplementary Material. *nifts* is freely available at https://github.com/cyhsuTN/nifts.

## Abbreviations

HR: hazard ratio
PH: proportional hazards
ITT: intention-to-treat
RCT: randomized controlled trials
NI: non-inferiority
RMST: restricted mean survival time
DRMST: difference in restricted mean survival times
RPSFTM: rank preserving structural accelerated failure time models

## References

[1] Uno H, Wittes J, Fu H, Solomon SD, Claggett B, Tian L, Alternatives to hazard ratios for comparing the efficacy or safety of therapies in noninferiority studies. Ann Intern Med. 2015;163:127–134. 26054047 10.7326/M14-1741 PMC4510023

[2] Cheng D, Pak K and Wei LJ. Demonstrating noninferiority of accelerated radiotherapy with panitumumab vs standard radiotherapy with cisplatin in locoregionally advanced squamous cell head and neck carcinoma. JAMA Oncol. 2017;3:1430–1431. 28750125 10.1001/jamaoncol.2017.0737 PMC7371341

[3] Kim DH, Uno H and Wei LJ. Restricted mean survival time as a measure to interpret clinical trial results. JAMA Cardiol. 2017;2:1179–1180. 28877311 10.1001/jamacardio.2017.2922 PMC6359932

[4] Irwin JO. The standard error of an estimate of expectation of life, with special reference to expectation of tumourless life in experiments with mice. Journal of Hygiene 1949;47:188–189. 15406758 10.1017/s0022172400014443 PMC2234920

[5] Royston P. and Parmar M.K.B. The use of restricted mean survival time to estimate the treatment effect in randomized clinical trials when the proportional hazards assumption is in doubt. Stat Med. 2011;30:2409–2421. 21611958 10.1002/sim.4274

[6] Royston P. and Parmar M.K.B. Restricted mean survival time: an alternative to the hazard ratio for the design and analysis of randomized trials with a time-to-event outcome. BMC Med Res Methodol. 2013;13:152. 24314264 10.1186/1471-2288-13-152 PMC3922847

[7] Freidlin B, Hu C, Korn EL. Are restricted mean survival time methods especially useful for Noninferiority Trials. Clin Trials. 2021;18:188–196. 33626896 10.1177/1740774520976576 PMC8329935

[8] Quartagno M. A comparison of different population-level summary measures for randomised trials with time-to-event outcomes, with a focus on non-inferiority trials. Clin Trials. 2023;20:594–602. 37337728 10.1177/17407745231181907 PMC7615295

[9] Weir IR, Trinquart L. Design of non-inferiority randomized trials using the difference in Restricted Mean Survival Times. Clin Trials. 2018;15: 499–508. 30074407 10.1177/1740774518792259 PMC6133762

[10] Phadnis MA, Mayo MS. Sample size calculations for noninferiority trials for time-to-event data using the concept of proportional time. J Appl Stat. 2020;48:1009–1032. 35707732 10.1080/02664763.2020.1753026 PMC9042171

[11] Van Cutsem E, Peeters M, Siena S, Humblet Y, Hendlisz A, Neyns B, Open-label phase III trial of panitumumab plus best supportive care compared with best supportive care alone in patients with chemotherapy refractory metastatic colorectal cancer. J Clin Oncol. 2007;25:1658–64. 17470858 10.1200/JCO.2006.08.1620

[12] Mo Y, Lim C, Watson JA, White NJ, Cooper BS. Non-adherence in non-inferiority trials: pitfalls and recommendations. BMJ. 2020;370:m2215. 32611541 10.1136/bmj.m2215 PMC7327542

[13] Whelan TJ, Pignol JP, Levine MN, Julian JA, MacKenzie R, Parpia S, Long-term results of hypofractionated radiation therapy for breast cancer. N Engl J Med. 2010;362:513–20. 20147717 10.1056/NEJMoa0906260

[14] Morden JP, Lambert PC, Latimer N, Abrams KR, Wailoo AJ. Assessing methods for dealing with treatment switching in randomised controlled trials: a simulation study. BMC Med Res Methodol. 2011;11:4. 21223539 10.1186/1471-2288-11-4 PMC3024998

[15] Deng L., Hsu C.-Y., Shyr Y. Power and sample sizes estimation in clinical trials with treatment switching in intention-to-treat analysis: a simulation study. BMC Med Res Methodol. 2023;23:49. 36823545 10.1186/s12874-023-01864-1 PMC9948351

[16] Pya N, Wood SN. Shape constrained additive models. Stat Comput. 2015; 25:543–55.

[17] Hsu C.-Y., Chen C.-H., Hsu K.-N., Lu Y.-H. A useful design utilizing the information fraction in a group sequential clinical trial with censored survival data. Biometrics. 2019;75:133–143. 30004574 10.1111/biom.12925

[18] Robins JM, Tsiatis AA. Correcting for non-compliance in randomized trials using rank preserving structural failure time models, Communications in Statistics - Theory and Methods. 1991;20:2609–2631.

